# Associations of Carbohydrate Intake With New-Onset Hypertension Subtypes: Results From the China Health and Nutrition Survey (2000–2011)

**DOI:** 10.3389/fnut.2021.728774

**Published:** 2022-01-06

**Authors:** Su-Fen Qi, Ya-Jing Cao, Hui-Jun Wang, Bing Zhang, Jing Yan, Qing-Bao Tian

**Affiliations:** ^1^Hebei Key Laboratory of Environment and Human Health, Department of Epidemiology and Statistics, School of Public Health, Hebei Medical University, Shijiazhuang, China; ^2^National Institute for Nutrition and Health, Chinese Center for Disease Control and Prevention, Beijing, China; ^3^Research Center of Electron Microscope, Hebei Medical University, Shijiazhuang, China

**Keywords:** carbohydrate intake, hypertension subtypes, China Health and Nutrition Survey, ISH, IDH

## Abstract

**Background:** The effects of carbohydrate intake on hypertension (HTN) subtypes are scarce. We examined the association of carbohydrate intake with new-onset HTN subtypes in Chinese adults.

**Methods:** Chinese Health and Nutrition Survey (CHNS) 2000–2011, 22,418 individuals were recorded using a 24-h recall method over three consecutive days. We excluded those who were pregnant women, lactating mothers, age <18 years, baseline age, blood pressure, and energy intake deficiency, extreme energy intake (male > 6,000 kcal or < 800 kcal; female > 4,000 kcal or < 600 kcal), and pulse pressure difference (Systolic Blood Pressure [SBP] - Diastolic Blood Pressure [DBP]) <10 mm Hg, HTN at baseline and data from only one survey. The total number of subjects who participated in at least two surveys was 7,930. The main outcome was new-onset HTN subtypes over 6.9 person years of follow-up.

**Results:** 2,521 participants were found to be HTN, which included 1,318 males (52.3%), 1,203 females (47.7%), 721 had systolic-diastolic hypertension (SDH, 28.6%), 655 had isolated systolic hypertension (ISH, 26.0%), and 993 had isolated diastolic hypertension (IDH, 39.4%). Compared with extreme quintiles of carbohydrate, multivariable-adjusted hazard ratios (HR) and 95% confidence intervals (CI) for new-onset HTN, SDH, ISH and IDH associated with carbohydrate intake were 1.12 (0.97–1.30), 1.54 (1.18–2.00), 0.89 (0.67–1.19) and 1.15 (0.91–1.45), respectively. The HR of SDH compared with extreme quintiles of carbohydrates was 1.56 (95% CI, 1.08–2.25; *P*_*trend*_ = 0.04) in men and 1.52 (95% CI, 1.02–2.26; *P*_*trend*_ = 0.02) in women.

**Conclusion:** Carbohydrates were related to a higher risk of SDH, which were not observed with HTN, ISH, and IDH.

## Introduction

Hypertension (HTN), as a major modifiable risk factor for cardiovascular disease, accounts for approximately 45% of global cardiovascular disease morbidity and mortality (1). Globally, the overall prevalence in adults ≥25 years of age was 40% in 2008, with the highest being 46% in Africa (2, 3). Previous studies in China have suggested that the prevalence of HTN in adults was from 14.5% in 1991 to 34.0% in 2012 (4–8). According to the China Health and Nutrition Survey (CHNS), the incidence of HTN is 4.4 per 100 person-years in the Chinese adults (9). Reducing the burden of diseases associated with HTN has been identified as a public health priority in the world as well as in China.

The causes of HTN include the intricate interplay between behavioral, environmental (including dietary factors), physiological, genetic, social, and economic factors (10). Evidence suggests that dietary factors are determinants of HTN (11). Some studies have found that a healthy and reasonable diet (such as the Mediterranean diet, Dietary Approaches to Stop Hypertension (DASH diet) can decrease blood pressure (12–14). Compared with Western countries, Chinese who eat carbohydrate-rich, low-fat diets have lower blood pressure levels, but the results are inconsistent. A cross-sectional study found that high-carbohydrate intake was associated with HTN by increasing inflammatory factors in rural areas in China (15). However, a prospective cohort study found that carbohydrate supply was not associated with the risk of developing HTN (16). There was no significant association between the risk of hypertension and intake of total carbohydrates (*P* = 0.9) among 80,426 French adults who participated in the NutriNet-Santé cohort study. In addition, studies have found that partial replacement of carbohydrates with protein or monounsaturated fat can lower blood pressure (17, 18). It was noteworthy that OmniHeart Randomized Trial extended previous knowledge about observations on the effects of protein and unsaturated fat on blood pressure. Compared with the carbohydrate diet, both protein and unsaturated fat diets significantly lowered systolic and diastolic blood pressure in all participants (17).

Few studies are investigating the relationship between carbohydrate intake and the incidence of HTN in China. However, most of them are cross-sectional studies and very few of them are large-scale prospective cohort studies. Since hypertension is associated with wide phenotypic variability, it can be divided into the following subtypes: isolated systolic hypertension (ISH), isolated diastolic hypertension (IDH), and systolic-diastolic hypertension (SDH) (19, 20). These subtypes may provide important information concerning the causation of hemodynamic and/or structural abnormalities that contribute to hypertension. No study has been found to investigate the relationship between HTN subtypes and carbohydrate intake. However, the dietary patterns and disease characteristics of the Chinese population are quite different from those of the Western population. Therefore, in this cohort study, the data of the China Health and Nutrition Survey (CHNS) from 2000 to 2011 was used to explore the relationship between carbohydrate intake and the risk of new incidence of HTN subtypes.

## Materials and Methods

### Study Design and Study Population

The CHNS is a representative sample and is the only large-scale longitudinal, household-based survey in China. This study was based on CHNS data completed in 2000, 2004, 2006, 2009, and 2011. In each survey, only adults aged ≥ 18 years and their data on sex, age, urban-rural status, body mass index (BMI), educational levels, lifestyle factors, and physical examinations (height, weight, as well as waist circumference) were extracted.

**Figure 1:**
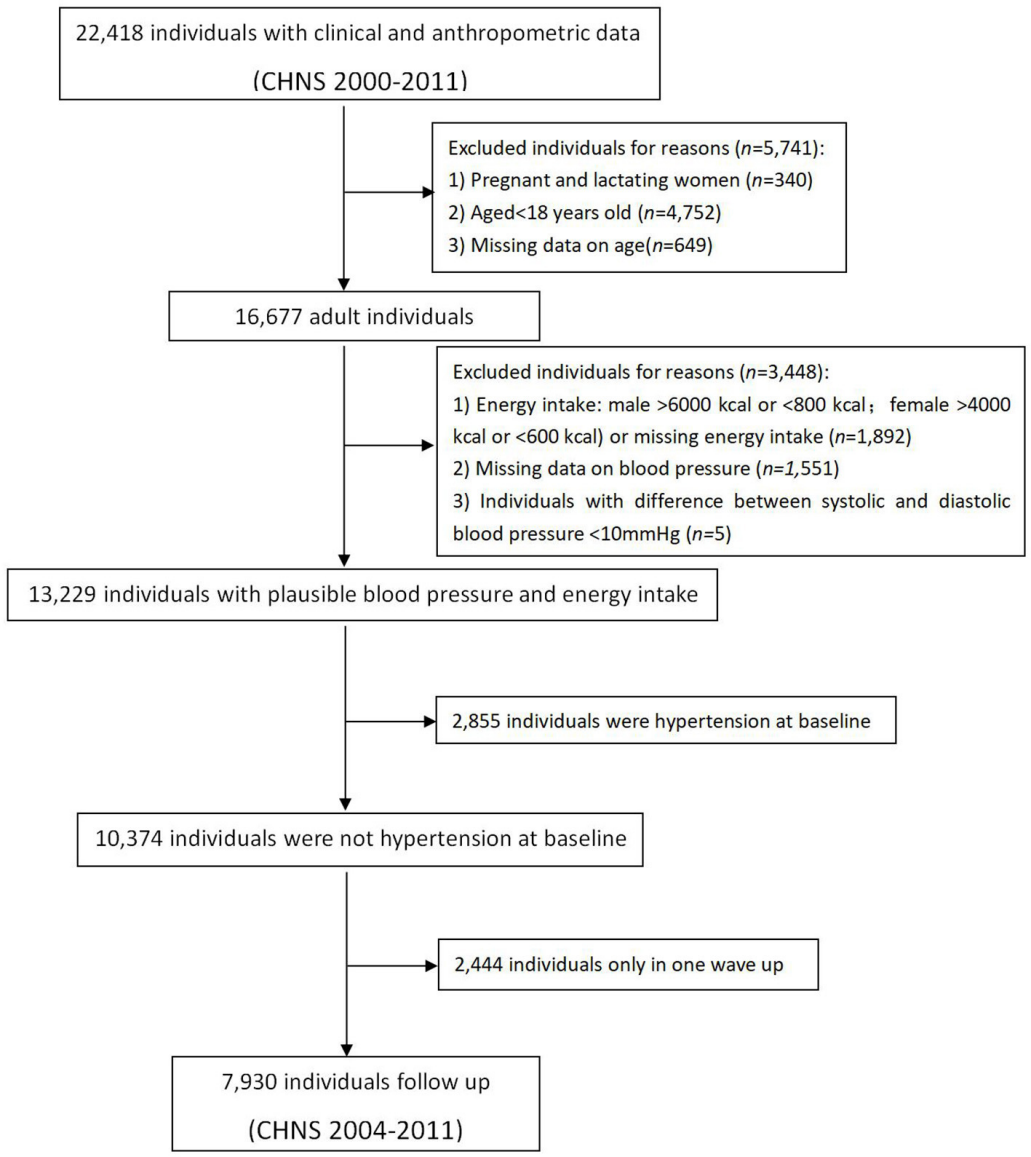
Flow chart illustrating the sample selection procedure.

To determine incident hypertension and subtypes, we identified a dynamic cohort study covering five time periods, which included the CHNS in 2000 and consequent follow-up surveys. The consequent follow-up surveys were considered as follow-up surveys of the former time period, and the surveys simultaneously added normotensive participants as new baseline surveys of follow-up time periods. To limit the biases caused by pre-existing factors, we excluded participants with ineligible factors in their baseline surveys, which is summarized in Figure 1. Pregnant or lactating women were excluded, along with incomplete data, blood pressure difference (Systolic Blood Pressure [SBP]–Diastolic Blood Pressure [DBP]) <10 mm Hg, implausible or extreme BMI (< 15.0 kg/m^2^ or > 40.0 kg/m^2^) or height (< 120.0 cm) or extreme or implausible energy intake (male > 6,000 kcal or < 800 kcal; female > 4,000 kcal or < 600 kcal). All the participants were free of HTN at baseline.

According to the inclusion exclusion criteria, a total of 7,930 subjects (free of HTN) participated in at least two surveys, including 3,744 males and 4,186 females. The process of this study is summarized in a flow chart in Figure 1.

### Calculation of Follow-Up Time

We calculated the IRs (per 100 person-years) of hypertension and subtypes according to the number of person-years of follow-up. For participants who did not develop hypertension at a later follow-up survey, the follow-up time was calculated from the date of the baseline survey to the date of later survey of follow-up. For participants who developed hypertension, the date of the onset of hypertension was assumed to be the midpoint between the two surveys because of the insidious onset of hypertension. For participants who did not complete the follow-up survey, the loss-to-follow-up date was also assumed to be the midpoint between the two surveys. Thus, the follow-up time was estimated to be the entire time during which the subjects remained free of hypertension plus half of the follow-up time during which hypertension developed or they were lost in the follow-up survey. This is a common method to deal with missing values (21).

### Definition of Hypertension and Subtypes

According to the 1999 regulations by the World Health Organization (WHO) (22) and 2018 Chinese Guidelines for Prevention and Treatment of Hypertension (23), hypertension is defined as: SBP ≥ 140 mm Hg and/or DBP ≥ 90 mm Hg and/or a history of hypertension, or currently using antihypertensive drugs. According to the mechanism of hypertension, hypertension is divided into three subtypes: (1) Isolated Systolic Hypertension (ISH), defined as SBP ≥140 mm Hg and DBP <90 mm Hg; (2) simple Isolated Diastolic Hypertension (IDH), defined as SBP <140 mm Hg and DBP ≥90 mm Hg; (3) Systolic-Diastolic Hypertension (SDH), defined as SBP ≥ 140 mm Hg and DBP ≥ 90 mm Hg.

### Dietary Assessment

Dietary assessment was based on three consecutive 24-h dietary recalls and a household-level food inventory record during the same time. The dietary data of the CHNS were obtained through a three-day (two weekdays and one weekend day), consecutive 24-h dietary recalls in each wave of the CHNS and were collected by trained investigators who weighed all the available foods in the participants' homes. Household food consumption was assessed by weighing all food consumed by the household (including cooking oil, salt, and other condiments) from the beginning to the end of each day during the survey period. Food stock at the initiation of the survey, food purchases, and/or production and food waste during the survey period were weighed and included in the calculation of household food consumption. In each survey cycle, individual dietary intake was assessed *via* three consecutive 24-hour dietary recalls (24), which is a validated method for assessing dietary intake (25, 26). Days for 24-h dietary recalls were randomly selected from Monday to Sunday and included at least 1 weekend day. All eligible participants were asked to follow their typical daily dietary patterns. All household members reported all detailed food items consumed at home and away from home (i.e., food items consumed at restaurants, canteens, and other locations) to well-trained interviewers during the same survey period. Interviewers recorded the type and amount of all food items consumed, as well as the type of meal and location of food consumption during the 24 h of the previous day. The amount of food in each dish was estimated from the household inventory, and the proportion of each dish consumed was reported by each person. Data for soft drink, sugared-sweetened fruit drink, and alcohol consumption during the past year were obtained by a food frequency questionnaire with five categories (almost every day, three to four times per week, once or twice per week, once or twice per month, or less than once per month). More details about dietary data have been reported elsewhere (27). Dietary assessment was the same in all surveys.

To reduce recall bias, self-report forms of dietary records were distributed to household units to help the participants record daily food intake and to serve as supplementary materials for formal dietary surveys. The quantity of cooking oil and condiments were simultaneously measured before and after 3 days of household investigation, and the quantities of purchases and waste were noted when entering the household every day. The amount of cooking oil and condiments that was consumed was obtained by weighing food and was divided according to the energy intake of other foods of each family member. The average daily dietary nutrient intake of each family member was calculated by using the three-day consecutive 24-h diet reviewed survey data and the proportionally distributed household cooking oil and condiments consumption data combined with the Chinese Food Composition Table (2002 and 2004 editions). Calculation of the mean average daily nutrients intake per person according to the Chinese Food Composition Table (FCTS) (28): including energy, carbohydrates, protein, fat, dietary fiber, vitamins, etc., and calculate carbohydrate percentage of energy intake (% E) = {([carbohydrate (G) x 4] / total energy intake [kcal]) x 100}.

### Covariates

For this cohort study, we also assessed hypertension-related factors: age, urban sites, education level, waist circumference, physical activities, smoke status, alcohol drinking status, personal income, and total energy intake.

Measuring equipment was provided by the project team; blood pressure, height, weight, and waist circumference were measured by trained health workers following the WHO's standardized program. Blood pressure (BP) was based on the mean of 3 consecutive measurements collected after 10 min of seated rest using standard mercury sphygmomanometers (22). The bodyweight of participants dressed in light clothing was measured without shoes to the nearest 0.1 kg with a calibrated beam scale (Seca North America 260). The height of barefoot subjects was measured to the nearest 0.1 cm using a portable stadiometer (Seca North America 880). The body mass index (BMI) was calculated as: BMI = weight (kg) / [height (m)^2^]. The result of waist circumference was the average of the two measurements. Urbanization was categorized as urban and rural. Education level was categorized as low-level (illiterate and primary school degree), middle-level (lower middle school degree) and high-level (upper–middle school degree, technical/vocational degree, university or college degree and master's degree or higher). Total energy intake was calculated from three-day dietary-recall food composition tables. The metabolic equivalent index (MET) codes and detailed questions of the physical activity survey were also described before (29). Smoking status was categorized as either ever or never smoked. Alcohol consumption status was categorized as either drinker or abstainer. Personal income questions summarized each part of an individual's income listed in the questionnaire. The study was approved by the ethics review committees of the Chinese Center for Disease Control and Prevention and University of North Carolina at Chapel Hill. Written informed consent was obtained from all participants. This study was registered at ClinicalTrials.gov (NCT04104308).

### Statistical Analysis

Continuous and categorical variables were described as means (95% confidence intervals [CIs]) and percentages (95% CIs). Overall, participants were categorized into five groups by quintile according to the percentage of energy (E) provided by carbohydrates, which was the total daily energy intake divided by the carbohydrate's energy (E = ([carbohydrate (g) ×4]/total energy intake [kcal]) ×100). The interval of follow-up was defined as the time between baseline and the earliest moment when participants were diagnosed as HTN subtypes, lost to or unavailable for follow-up, or at the end of follow-up.

Hazard ratios (HRs) and 95% CIs of HTN subtypes were calculated in accordance with individual carbohydrate intake (divided into five groups) in a time dependent Cox proportional hazards regression model with follow-up duration as the time scale. To quantify the trend, a median in each quintile was assigned and modeled constantly with statistical significance checked using the Wald test.

Subgroup analyses with multiplicative interaction terms were performed to show whether the associations of carbohydrate intake levels with the risk of HTN subtypes varied by age (<50 or ≥ 50 y), urban sites (yes or no), educational levels (low or high), waist circumference (<80 or ≥ 80 cm in women; <85 or ≥ 85 cm in men), BMI (<24 or ≥24 kg/m^2^), energy intake (<2,100 or ≥ 2,100 kcal in women; <2,400 or ≥ 2,400 kcal in men), physical activity (<200 or ≥ 200 METs-hour/week), smoking status (ever or never), and alcohol consumption status (drinker or abstainer). *P* value was determined using the Wald test.

Statistical analyses were done with SPSS, version 20.0. All *P* values were 2-tailed; *P* < 0.05 was considered statistically significant.

## Results

### Characteristics of Participants at Baseline

The mean SBP and DBP were 116.2 (115.9–116.5) mm Hg, 75.8 (74.5–76.0) mm Hg for men, and 112.7 (112.3, 113.1) mm Hg, 73.5 (73.2, 73.7) mm Hg for women at baseline (Tables 1, 2). The average baseline energy intake was 2,455.9 (2,435.0–2,476.9) kcal/d for men and 2,077.3 (2,060.8–2,093.9) kcal/d for women. The average intake of carbohydrates was 357.0 (353.3–360.7) g/d for men and 300.3 (297.4–303.2) g/d for women (Tables 1, 2).

**Table 1:** Characteristics of the study participants at the baseline (*n* = 7,930).

**Variable**	**Quintile of Percentage energy from carbohydrate**					**Total**
	**Quintile 1**	**Quintile 2**	**Quintile 3**	**Quintile 4**	**Quintile 5**	
Age (years)^a^	45.1 (44.4–45.8)	43.7 (43.0–44.3)	43.2 (42.6–43.9)	42.8 (42.1–43.4)	41.7 (41.0–42.3)	43.3 (43.0–43.6)
Male (%)^b^	47.5 (45.1–50.0)	44.9 (42.5–47.3)	45.5 (43.0–47.9)	48.9 (46.5–51.4)	49.2 (46.8–51.7)	47.2 (46.1–48.3)
Urban location (%)^b^	56.3 (53.8–58.7)	44.5 (42.0–46.9)	34.2 (31.8–36.5)	21.8 (19.8–23.8)	14.2 (12.5–16.0)	34.2 (33.1–35.2)
SBP (mmHg)^a^	115.2 (114.6–115.7)	114.6 (114.0–115.1)	114.2 (113.6–114.7)	114.2 (113.7–114.8)	113.6 (113.0–114.2)	114.4 (114.1–114.6)
DBP (mmHg)^a^	75.3 (74.9–75.7)	74.7 (74.3–75.1)	74.6 (74.2–74.9)	74.3 (73.9–74.7)	74.0 (73.6–74.4)	74.6 (74.4–74.7)
Waist (cm)^a^	80.2 (79.7–80.7)	79.2 (78.7–79.6)	78.5 (78.0–78.9)	78.4 (77.9–78.8)	78.1 (77.7–78.6)	78.9 (78.7–79.1)
BMI (kg/m^2^)^a^	22.9 (22.7–23.0)	22.5 (22.3–22.7)	22.4 (22.2–22.6)	22.3 (22.1–22.5)	22.1 (22.0–22.3)	22.4 (22.4–22.5)
Ever smoking (%)^b^	31.7 (29.4–34.0)	30.0 (27.8–32.3)	29.9 (27.6–32.1)	32.5 (30.2–34.8)	32.1 (29.8–34.4)	31.3 (30.2–32.3)
Alcohol intake (%)^b^	39.3 (36.9–41.7)	33.2 (30.9–35.6)	32.2 (29.9–34.5)	32.7 (30.4–35.0)	32.5 (30.2–34.8)	34.0 (32.9–35.0)
**Education levels**						
Primary/illiterate (%)^b^	30.7 (28.41–33.0)	32.8 (30.4–35.1)	39.2 (36.8–41.6)	44.7 (42.3–47.2)	51.7 (49.2–54.2)	39.8 (38.7–40.9)
Middle school (%)^b^	31.5 (29.2–33.8)	34.3 (31.9–36.6)	34.5 (32.2–36.8)	34.2 (31.8–36.5)	33.4 (31.0–35.7)	33.6 (32.5–34.6)
High/above (%)^b^	35.6 (33.3–38.0)	31.6 (29.3–33.8)	25.2 (23.0–27.3)	19.0 (17.1–20.9)	12.8 (11.2–14.4)	24.8 (23.9–25.8)
Physical Activity (MET–h/wk)^a^	223.3 (213.7–232.8)	253.8 (243.2–264.4)	294.5 (282.9–306.1)	350.4 (338.2–362.6)	402.8 (391.6–414.1)	304.9 (299.7–310.1)
Income (yuan/year)^a^	4787.6 (4191.8–5383.4)	3591.3 (3184.2–3998.5)	2967.9 (2549.9–3385.9)	1842.9 (1609.6–2076.3)	1288.1 (970.3–1605.9)	2895.3 (2709.0–3081.6)
Energy intake (kcal/d)^a^	2339.7 (2305.9–2373.6)	2270.0 (2240.1–2299.8)	2243.7 (2213.8–2273.6)	2220.1 (2191.0–2249.2)	2207.0 (2175.6–2238.4)	2256.1 (2242.3–2269.9)
Carbohydrate intake (g/d)^a^	246.0 (242.1–249.8)	297.1 (293.1–301.0)	328.4 (324.0–332.7)	358.8 (354.1–363.5)	405.0 (399.1–410.9)	327.1 (324.7–329.4)
Fat intake (g/d)^a^	111.4 (109.5–113.4)	87.1 (85.8–88.3)	72.2 (71.1–73.3)	58.2 (57.3–59.1)	37.9 (37.2–38.6)	73.4 (72.6–74.2)
Protein intake (g/d)a	75.5 (74.1–77.0)	70.2 (69.1–71.3)	67.3 (66.3–68.4)	64.1 (63.2–65.1)	61.1 (60.1–62.1)	67.7 (67.1–68.2)

a *Mean with 95% confidence interval (95% CI)*.

b *Percentage with 95% confidence interval (95% CI)*.

*MET, metabolic equivalents*.

**Table 2:** Characteristics of the study participants at the baseline among Chinese men and women.

**Variable**	**Quintile of Percentage energy from carbohydrate (Men)**					**Quintile of Percentage energy from carbohydrate (Women)**					**Total**
	**Quintile 1**	**Quintile 2**	**Quintile 3**	**Quintile 4**	**Quintile 5**	**Quintile 1**	**Quintile 2**	**Quintile 3**	**Quintile 4**	**Quintile 5**	
Age (years)^a^	45.2 (44.2–46.2)	43.6 (42.6–44.7)	43.1 (42.1–44.1)	41.9 (40.9–42.9)	41.0 (40.0–41.9)	44.9 (43.9–45.9)	44.0 (43.0–44.9)	43.2 (42.3–44.1)	43.5 (42.6–44.3)	42.5 (41.6–43.4)	43.0 (42.5–43.4)
Urban location (%)^b^	55.7 (52.2–59.3)	42.6 (39.1–46.2)	34.1 (30.7–37.5)	22.3 (19.3–25.3)	12.8 (10.4–15.2)	56.5 (53.2–59.9)	45.7 (42.3–49.1)	35.4 (32.2–38.7)	20.6 (17.8–23.3)	15.7 (13.3–18.2)	33.5 (32.0–35.0)
SBP (mmHg)^a^	117.2 (116.4–118.0)	116.2 (115.4–116.9)	116.1 (115.3–116.8)	115.8 (115.0–116.5)	115.8 (115.0–116.6)	113.3 (112.5–114.1)	113.3 (112.5–114.1)	112.5 (111.8–113.3)	113.0 (112.2–113.8)	111.4 (110.6–112.2)	116.2 (115.9–116.5)
DBP (mmHg)^a^	76.8 (76.3–77.3)	76.3 (75.7–76.8)	75.6 (75.1–76.2)	75.3 (74.8–75.9)	74.8 (74.2–75.4)	73.9 (73.3–74.4)	73.5 (72.9–74.0)	73.6 (73.0–74.1)	73.4 (72.8–74.0)	73.1 (72.6–73.7)	75.8 (74.5–76.0)
Waist (cm)^a^	82.8 (82.1–83.5)	81.2 (80.6–81.9)	80.2 (79.6–80.9)	79.3 (78.7–80.0)	79.3 (78.7–80.0)	77.8 (77.2–78.4)	77.5 (76.9–78.1)	77.2 (76.5–77.8)	77.1 (76.5–77.7)	77.0 (76.4–77.6)	80.6 (80.3–80.9)
BMI (kg/m^2^)^a^	23.0 (22.7–23.2)	22.5 (22.2–22.7)	22.3 (22.1–22.6)	22.0 (21.7–22.2)	22.1 (21.8–22.3)	22.8 (22.5–23.0)	22.5 (22.2–22.8)	22.5 (22.3–22.7)	22.6 (22.3–22.8)	22.2 (22.0–22.5)	22.4 (22.4–22.5)
Ever smoking (%)^b^	62.4 (59.0–65.9)	61.1 (57.6–64.6)	62.9 (59.5–66.4)	61.0 (57.5–64.5)	61.5 (58.1–65.0)	3.9 (2.6–5.3)	4.4 (3.0–5.8)	2.4 (1.4–3.4)	4.5 (3.1–6.0)	4.3 (2.9–5.7)	61.8 (60.3–63.4)
Alcohol intake (%)^b^	67.9 (64.6–71.3)	59.2 (55.7–62.7)	61.5 (58.0–64.9)	57.9 (54.4–61.5)	57.9 (54.4–61.5)	13.4 (11.1–15.7)	12.1 (9.9–14.3)	8.5 (6.6–10.4)	7.7 (5.9–9.5)	8.0 (6.2–9.8)	60.9 (59.3–62.5)
**Education levels**											
Primary/illiterate (%)^b^	26.1 (22.9–29.2)	24.2 (21.1–27.3)	32.7 (29.3–36.0)	34.7 (31.3–38.1)	41.8 (38.3–45.3)	34.9 (31.7–38.1)	39.8 (36.5–43.2)	42.8 (39.5–46.2)	54.8 (51.4–58.2)	62.0 (58.7–65.3)	31.9 (30.4–33.4)
Middle school (%)^b^	33.4 (30.0–36.8)	38.9 (35.4–42.4)	38.3 (34.8–41.8)	40.3 (36.8–43.8)	39.5 (36.0–43.0)	29.9 (26.8–33.0)	30.1 (27.0–33.3)	33.2 (30.0–36.4)	27.9 (24.8–30.9)	26.5 (23.5–29.4)	38.1 (36.5–39.6)
High/above (%)^b^	39.2 (35.7–42.7)	35.4 (32.0–38.9)	28.0 (24.8–31.2)	23.1 (20.1–26.1)	17.2 (14.5–19.9)	32.4 (29.2–35.5)	28.3 (25.3–31.4)	22.8 (20.0–25.6)	15.0 (12.5–17.4)	8.8 (6.9–10.7)	28.6 (27.1–30.0)
Physical Activity (MET–h/wk)^a^	227.5 (213.5–241.4)	257.1 (241.8–272.4)	295.7 (278.7–312.7)	346.4 (329.5–363.2)	391.8 (375.7–407.9)	219.8 (206.7–232.9)	252.9 (238.2–267.6)	287.4 (271.8–303.1)	357.3 (339.8–374.7)	411.7 (395.9–427.5)	303.7 (296.4–311.1)
Income (yuan/year)^a^	6454.8 (5313.0–7596.6)	4753.9 (4028.8–5479.0)	3940.6 (3213.7–4667.5)	2531.9 (2125.0–2938.7)	2035.1 (1387.4–2682.8)	3293.2 (2833.1–3753.3)	2638.5 (2181.2–3095.9)	2173.8 (1766.0–2581.7)	1095.5 (877.5–1313.5)	595.4 (445.6–745.2)	3942.4 (3596.2–4288.5)
Energy intake (kcal/d)^a^	2542.6 (2490.9–2594.3)	2484.4 (2439.4–2529.4)	2458.3 (2414.5–2502.0)	2542.6 (2490.9–2594.3)	2484.4 (2439.4–2529.4)	2155.4 (2114.6–2196.2)	2099.6 (2062.9–2136.3)	2061.3 (2026.0–2096.6)	2052.6 (2018.1–2087.1)	2017.9 (1981.2–2054.6)	2455.9 (2435.0–2476.9)
Carbohydrate intake (g/d)^a^	265.4 (259.5–271.2)	326.4 (320.4–332.4)	362.1 (355.7–368.6)	390.1 (382.7–397.5)	440.7 (431.8–449.7)	228.3 (223.6–233.1)	273.8 (269.0–278.6)	299.9 (294.8–305.0)	330.0 (324.4–335.6)	369.3 (362.4–376.3)	357.0 (353.3–360.7)
Fat intake (g/d)^a^	117.5 (114.4–120.6)	93.3 (91.4–95.2)	77.2 (75.6–78.8)	61.3 (59.9–62.7)	40.5 (39.3–41.6)	105.9 (103.4–108.3)	82.2 (80.6–83.9)	68.1 (66.8–69.4)	55.1 (54.0–56.1)	35.2 (34.3–36.1)	77.9 (76.7–79.1)
Protein intake (g/d)^a^	81.3 (79.0–83.5)	76.0 (74.3–77.8)	73.6 (72.1–75.2)	69.7 (68.2–71.1)	66.4 (64.8–67.9)	70.4 (68.6–72.3)	65.4 (64.0–66.9)	61.7 (60.4–63.0)	59.2 (58.0–60.3)	55.9 (54.7–57.0)	73.4 (72.6–74.2)

a *Mean with 95% confidence interval (95% CI)*.

b *Percentage with 95% confidence interval (95% CI).MET=metabolic equivalents*.

### Incidence Rates of Hypertension and Subtypes

**Figure 2:**
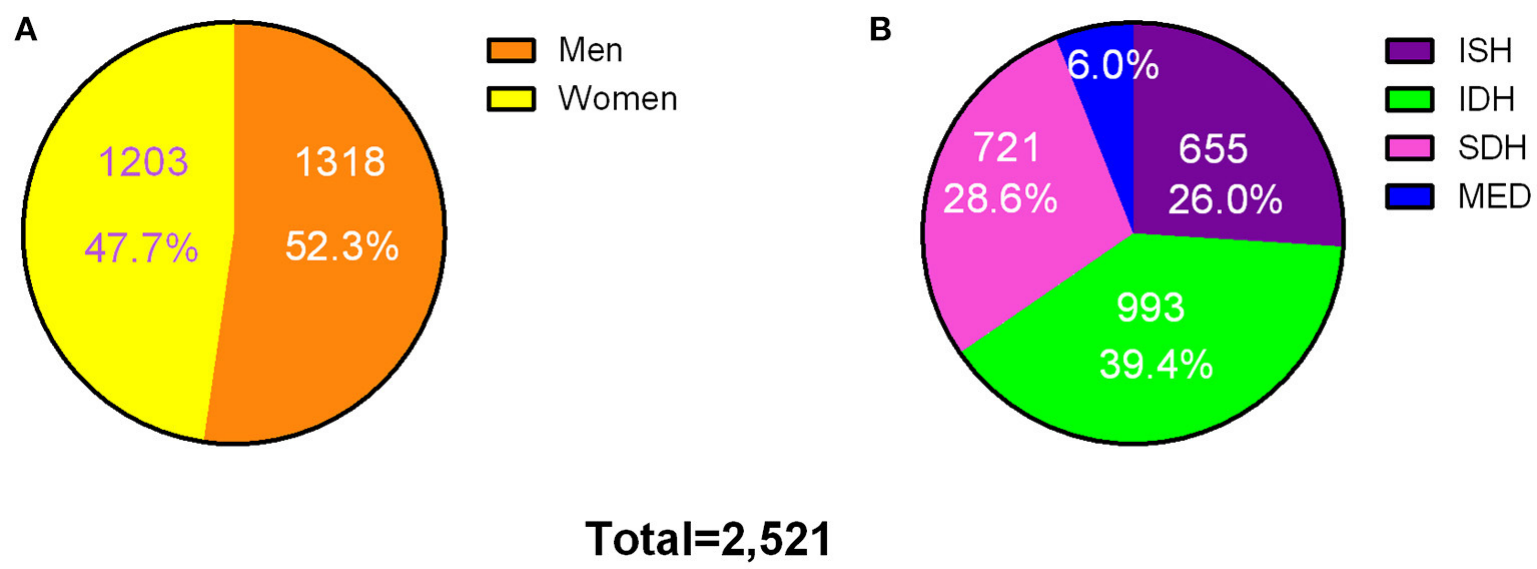
Incident cases of hypertension and subtypes among the participants. Incident cases of hypertension by gender **(A)** and subtypes **(B)**. ISH, isolated systolic hypertension; IDH, isolated diastolic hypertension; SDH, systolic-diastolic hypertension; MED, currently using antihypertensive medication.

The average follow-up time of this study was 7.3 person-years. During the follow-up, 2,521 people were found to have hypertension (HTN), including 1,318 (52.3%) males and 1,203 females (47.7%), 655 had ISH (26.0%), 993 had IDH (39.4%), and 721 had SDH (28.6%) (Figure 2). The age-adjusted incidence of HTN was 4.86 (95% CI, 4.68-5.04)/100 person-years, 5.48 (95% CI, 5.20–5.76)/100 person-years for males and 4.31 (95% CI, 4.08–4.55)/100 person-years for females (Table 3). The age-adjusted morbidity rates of ISH, IDH, and SDH were 1.48 (95% CI, 1.38–1.59)/100 person-years, 1.69 (95% CI, 1.59–1.80)/100 person-years and 1.38 (95% CI, 1.28–1.47)/100 person-years (Table 3). The incidence of SDH increased with the increase of carbohydrate energy intake (*P*_trend_ < 0.05), which was not observed in HTN, ISH, and IDH in men or women (Table 3).

**Table 3:** Incident rates of hypertension and subtypes in participants by quantiles of percentage energy from carbohydrate.

**Variable**	Incidence (per 100 person–years; 95% CI)^*^					**Total**
	**Quintile 1**	**Quintile 2**	**Quintile 3**	**Quintile 4**	**Quintile 5**	
HTN						
All						
Median (IQR)	43.7 (39.3–46.5)	52.4 (50.7–54.0)	58.5 (57.1–60.1)	64.5 (63.1–66.1)	72.7 (70.2–75.9)	58.5 (50.7–66.1)
Cases/n	475	494	474	538	540	2521
Person–years	9997.7	10330.8	11129.3	11454.1	11549.7	54461.6
IR	4.71 (4.30–5.13)	5.00 (4.58–5.42)	4.66 (4.27–5.05)	4.92 (4.53–5.32)	5.13 (4.73–5.54)	4.86 (4.68–5.04)
Men						
Median (IQR)	43.4 (39.0–46.4)	52.6 (51.0–54.3)	59.0 (57.5–60.4)	64.9 (63.6–66.6)	72.8 (70.5–76.2)	59.0 (50.9–66.6)
Cases/n	257	262	258	262	279	1318
Person–years	4567.2	4813.7	5163.7	5279.0	5291.6	25115.2
IR	5.83 (5.15–6.51)	5.52 (4.87–6.16)	5.43 (4.82–6.05)	5.32 (4.72–5.93)	5.49 (4.87–6.10)	5.48 (5.20–5.76)
Women						
Median (IQR)	44.0 (39.6–46.7)	52.2 (50.6–53.8)	58.2 (56.7–59.8)	64.2 (62.8–65.8)	72.6 (70.0–75.8)	58.2 (50.6–65.8)
Cases/n	217	238	217	269	262	1203
Person–years	5444.3	5520.9	5911.7	6183.5	6253.8	29346.3
IR	4.03 (3.51–4.56)	4.61 (4.06–5.16)	4.12 (3.61–4.62)	4.81 (4.27–5.34)	4.82 (4.29–5.35)	4.31 (4.08–4.55)
ISH						
All						
Cases/n	130	130	137	129	129	655
Person–years	9997.7	10330.8	11129.3	11454.1	11549.7	54461.6
IR	1.39 (1.16–1.62)	1.51 (1.28–1.75)	1.58 (1.35–1.81)	1.43 (1.22–1.65)	1.59 (1.36–1.82)	1.48 (1.38–1.59)
Men						
Cases/n	61	51	64	50	57	283
Person–years	4567.2	4813.7	5163.7	5279.0	5291.6	25115.2
IR	1.57 (1.21–1.93)	1.23 (0.92–1.54)	1.66 (1.31–2.01)	1.27 (0.97–1.58)	1.43 (1.11–1.75)	1.42 (1.28–1.57)
Women						
Cases/n	68	79	73	80	72	372
Person–years	5444.3	5520.9	5911.7	6183.5	6253.8	29346.3
IR	1.36 (1.05–1.67)	1.77 (1.42–2.11)	1.60 (1.28–1.92)	1.61 (1.30–1.93)	1.66 (1.34–1.97)	1.53 (1.39–1.67)
IDH						
All						
Cases/n	181	194	186	207	225	993
Person–years	9997.7	10330.8	11129.3	11454.1	11549.7	54461.6
IR	1.69 (1.44–1.95)	1.81 (1.56–2.07)	1.56 (1.33–1.79)	1.58 (1.35–1.81)	1.82 (1.58–2.07)	1.69 (1.59–1.80)
Men						
Cases/n	111	113	118	118	126	586
Person–years	4567.2	4813.7	5163.7	5279.0	5291.6	25115.2
IR	2.35 (1.91–2.79)	2.23 (1.82–2.65)	2.10 (1.71–2.49)	1.98 (1.60–2.35)	2.23 (1.83–2.62)	2.16 (1.98–2.34)
Women						
Cases/n	70	83	75	83	96	407
Person–years	5444.3	5520.9	5911.7	6183.5	6253.8	29346.3
IR	1.19 (0.91–1.48)	1.46 (1.14–1.78)	1.21 (0.93–1.49)	1.24 (0.97–1.52)	1.44 (1.14–1.73)	1.29 (1.16–1.42)
SDH						
All						
Cases/n	116	143	122	176	164	721
Person–years	9997.7	10330.8	11129.3	11454.1	11549.7	54461.6
IR	1.16 (0.95–1.36)	1.36 (1.14–1.59)	1.21 (1.01–1.42)	1.63 (1.40–1.87)	1.53 (1.31–1.76)#	1.38 (1.28–1.47)
Men						
Cases/n	62	84	67	86	87	386
Person–years	4567.2	4813.7	5163.7	5279.0	5291.6	25115.2
IR	1.36 (1.02–1.69)	1.73 (1.36–2.10)	1.42 (1.09–1.74)	1.88 (1.52–2.25)	1.66 (1.32–2.01)#	1.60 (1.44–1.75)
Women						
Cases/n	54	60	54	85	82	335
Person–years	5444.3	5520.9	5911.7	6183.5	6253.8	29346.3
IR	1.04 (0.77–1.31)	1.04 (0.77–1.31)	1.04 (0.78–1.30)	1.49 (1.18–1.79)	1.54 (1.23–1.84)#	1.18 (1.06–1.30)

* *Age adjusted using the direct method to the year 2010 census population*.

*IR, incidence rate, per 100 person-years; CI, confidence interval*.

# *P for trend (P_trend_ < 0.05)*.

*ISH, isolated systolic hypertension; IDH, isolated diastolic hypertension; SDH, systolic-diastolic hypertension; HTN, hypertension*.

### Associations Between the Intake of Carbohydrates and Hypertension and Subtypes

**Figure 3:**
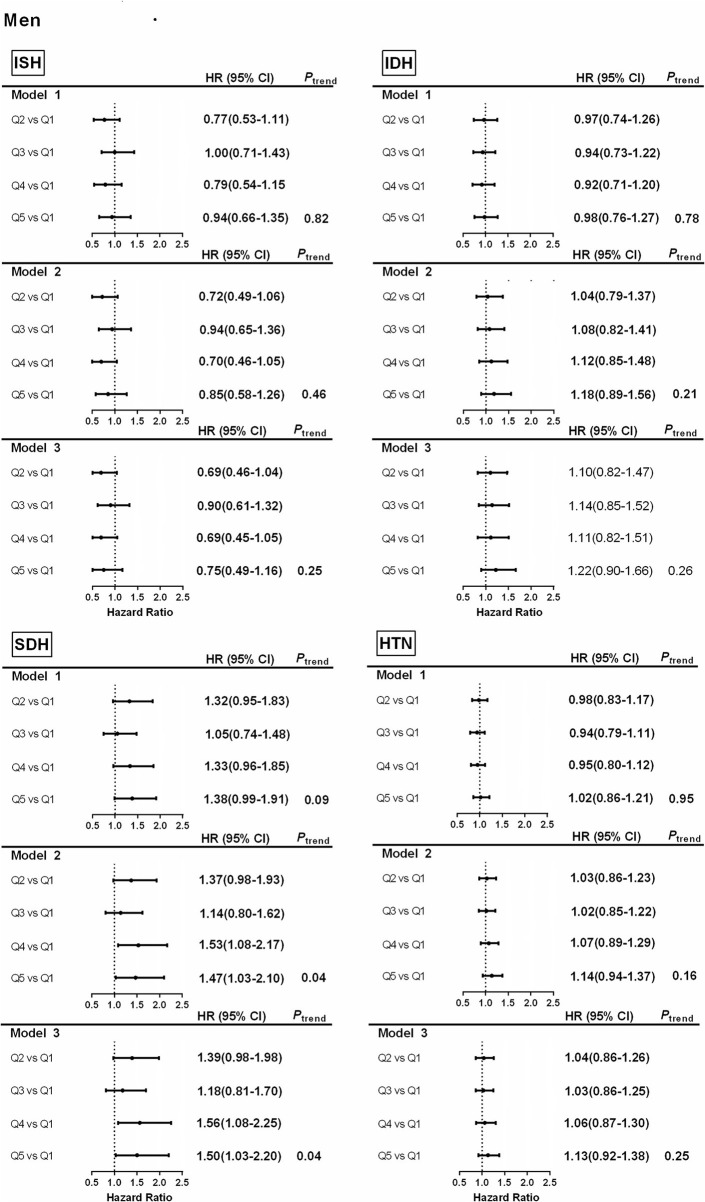
Associations between percentage energy from carbohydrate by quantiles and hypertension and subtypes in men. Model 1. Hazard ratios and 95% CIs are adjusted for age. Model 2. Hazard ratios and 95% CIs are adjusted for age, urban or rural location, education level, waist circumference, ever smoking (never, ever), alcohol drinking (abstainer or drinker), personal income, BMI (underweight, normal weight, overweight, and obesity). Model 3. Hazard ratios and 95% CIs are adjusted for age, urban or rural location, education level, waist circumference, ever smoking (never, ever), alcohol drinking (abstainer or drinker), personal income, BMI (underweight, normal weight, overweight, and obesity), physical activity and energy intake. ISH, isolated systolic hypertension; IDH, isolated diastolic hypertension; SDH, systolic–diastolic hypertension; HTN, hypertension.

**Figure 4:**
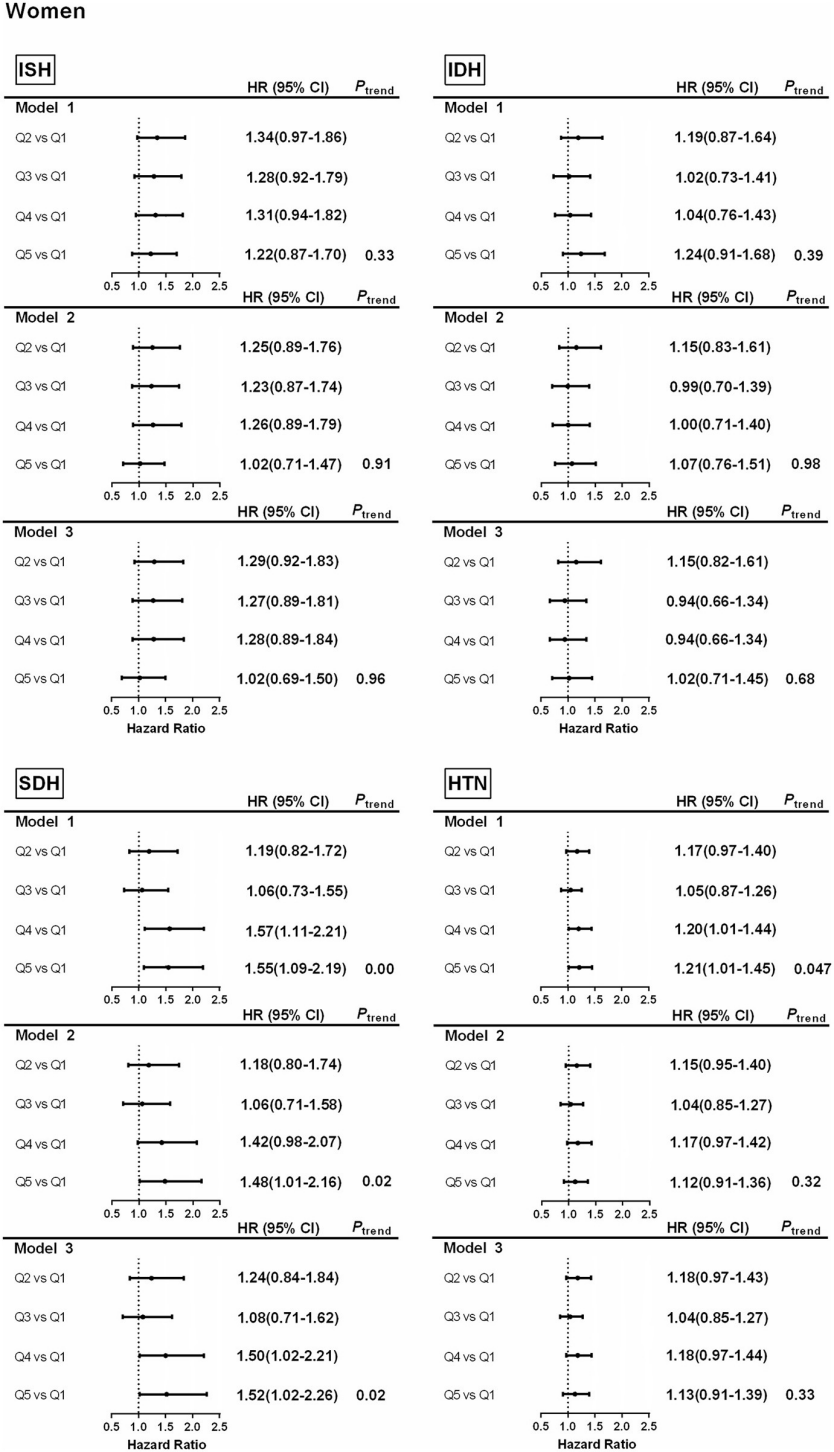
Associations between percentage energy from carbohydrate by quantiles and hypertension and subtypes in women. Model 1. Hazard ratios and 95% CIs are adjusted for age. Model 2. Hazard ratios and 95% CIs are adjusted for age, urban or rural location, education level, waist circumference, ever smoking (never, ever), alcohol drinking (abstainer or drinker), personal income, BMI (underweight, normal weight, overweight, and obesity). Model 3. Hazard ratios and 95% CIs are adjusted for age, urban or rural location, education level, waist circumference, ever smoking (never, ever), alcohol drinking (abstainer or drinker), personal income, BMI (underweight, normal weight, overweight, and obesity), physical activity and energy intake. ISH, isolated systolic hypertension; IDH, isolated diastolic hypertension; SDH, systolic–diastolic hypertension; HTN, hypertension.

Subjects were divided into five groups according to the quintile of baseline carbohydrate energy supply ratio: Quintile 1 (43.7%, 95% CI = 39.3–46.5%), Quintile 2 (52.4%, 95%CI = 50.7–54.0%), Quintile 3(58.5%, 95% CI = 57.1–60.1%), Quintile 4 (64.5%,95%CI = 63.1–66.1%), Quintile 5 (72.7%,95% CI = 70.2–75.9%). Higher carbohydrate intake was related to a higher risk of SDH in the potential confounding variables-adjusted models of men (quartile 5 vs. quartile 1, HR 1.50 (1.03–2.20), *P*_trend_ = 0.04) and women (quartile 5 vs. quartile 1, HR 1.52 (1.02–2.26), *P*_trend_ = 0.02) (Figures 3, 4), The results showed that the risk of SDH increased with the increase of carbohydrate However, it was not associated with HTN, ISH, and IDH (Figures 3, 4).

### Subgroup Analyses

**Figure 5:**
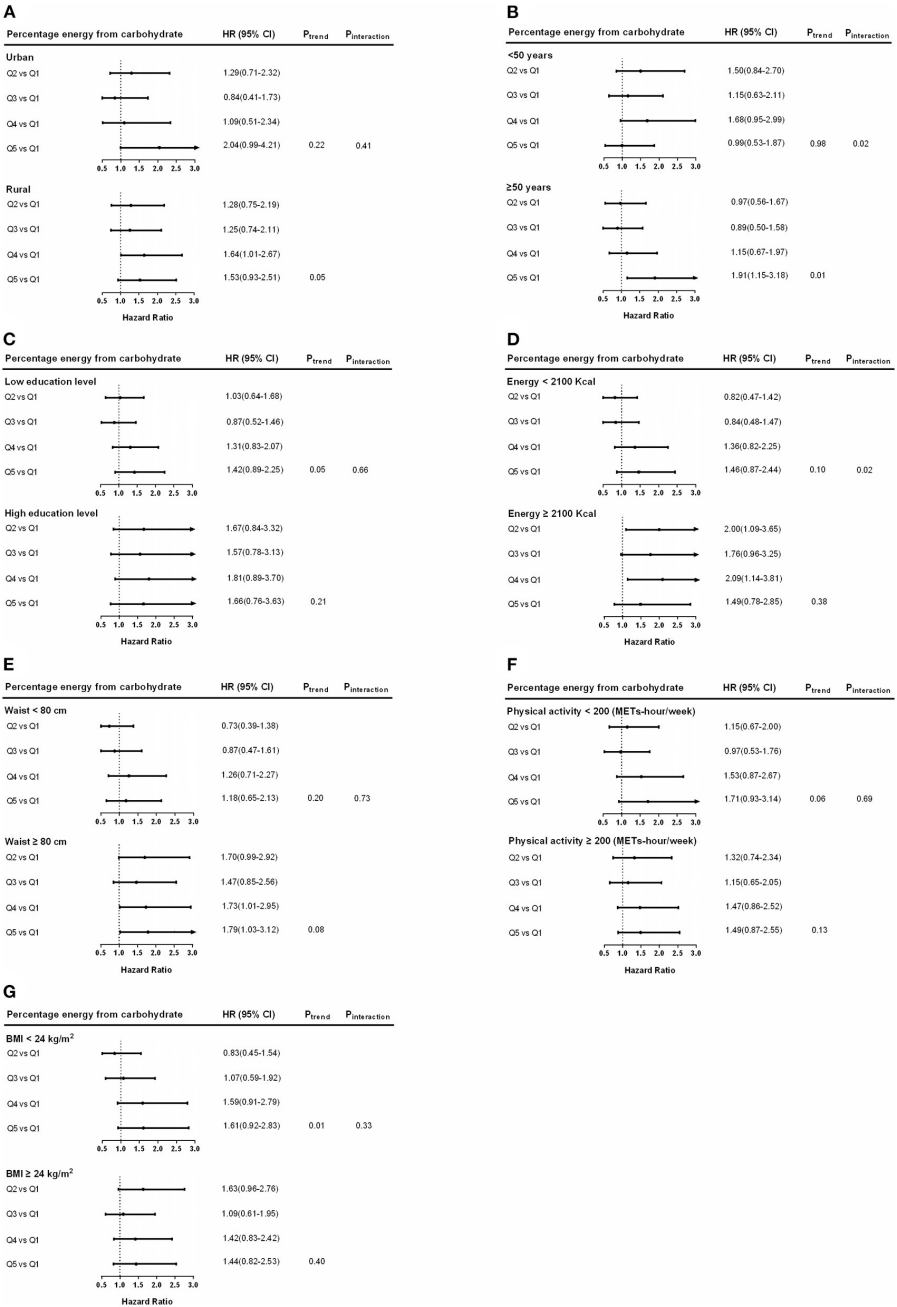
Hazard ratios (HRs) for SDH risk for percentage energy from carbohydrate in subgroups among women. Hazard ratios and 95% CIs are adjusted for age, urban or rural location, education, waist circumference, ever smoking (never, ever), alcohol drinking (abstainer or drinker), personal income, BMI (underweight, normal weight, overweight, and obesity), physical activity, and energy intake. Hazard ratios (95% CIs) of hypertension among women by region **(A)**, age **(B)**, education levels **(C)**, energy intake **(D)**, waist circumference **(E)**, physical activity **(F)**, and BMI **(G)** are shown. Ptrend, P for trend; Pinteraction, P for interaction.

To gain a further understanding of the relationship between the risk of hypertension and subgroups and the intake of carbohydrates, a subgroup analysis was carried out. In men, positive associations between carbohydrate intake and SDH were found in the individuals who lived in rural areas, had a higher educational level and lower energy intake (all *P*_trend_ < 0.05, *P*_interaction_ > 0.05) (Table 4). In women, positive associations between carbohydrate intake and SDH were found in older individuals (≥ 50 years) who lived in rural areas, had a higher energy intake, and a larger waist circumference (all *P*_trend_ < 0.05, *P*_interaction_ > 0.05) (Figure 5).

**Table 4:** Hazard ratios (95% CIs) for HTN and SDH risk for quintiles of carbohydrate in subgroups of men.

**Subgroups**	**HTN**							**SDH**						
	**Quintile 1**	**Quintile 2**	**Quintile 3**	**Quintile 4**	**Quintile 5**	***P_***trend***_***	***P_***interaction***_***	**Quintile 1**	**Quintile 2**	**Quintile 3**	**Quintile 4**	**Quintile 5**	***P_***trend***_***	***P_***interaction***_***
Region Urban	1 (Reference)	1.03 (0.78–1.37)	0.99 (0.73–1.33)	1.09 (0.77–1.53)	1.30 (0.86–1.96)	0.35	0.81	1 (Reference)	1.18 (0.71–1.99)	0.96 (0.54–1.70)	1.38 (0.76–2.51)	1.03 (0.44–2.41)	0.62	0.88
Rural	1 (Reference)	1.03 (0.80–1.33)	1.04 (0.81–1.34)	1.03 (0.80–1.32)	1.07 (0.84–1.38)	0.61		1 (Reference)	1.65 (0.99–2.76)	1.42 (0.84–2.39)	1.76 (1.06–2.93)	1.76 (1.06–2.93)	0.06	
Age < 50 years	1 (Reference)	1.07 (0.83–1.37)	1.00 (0.77–1.29)	1.09 (0.84–1.42)	1.21 (0.93–1.57)	0.17	0.67	1 (Reference)	1.32 (0.82–2.12)	0.97 (0.59–1.62)	1.45 (0.89–2.35)	1.44 (0.87–2.38)	0.17	0.84
≥ 50 years	1 (Reference)	1.03 (0.77–1.36)	1.03 (0.78–1.37)	1.00 (0.74–1.36)	0.90 (0.65–1.25)	0.56		1 (Reference)	1.47 (0.86–2.51)	1.33 (0.78–2.29)	1.53 (0.88–2.69)	1.34 (0.74–2.40)	0.39	
Education levels Low	1 (Reference)	0.98 (0.70–1.37)	1.06 (0.77–1.45)	0.92 (0.66–1.27)	1.11 (0.81–1.52)	0.60	0.71	1 (Reference)	1.90 (1.03–3.47)	1.55 (0.84–2.85)	1.17 (0.62–2.22)	1.76 (0.96–3.23)	0.41	0.06
High	1 (Reference)	1.08 (0.86–1.36)	1.02 (0.80–1.30)	1.16 (0.90–1.49)	1.14 (0.87–1.49)	0.29		1 (Reference)	1.21 (0.78–1.88)	1.00 (0.62–1.62)	1.89 (1.20–2.96)	1.35 (0.80–2.26)	0.06	
Energy intake < 2,400 kcal	1 (Reference)	1.01 (0.77–1.33)	1.09 (0.83–1.43)	1.00 (0.83–1.32)	1.09 (0.82–1.45)	0.64	0.82	1 (Reference)	1.40 (0.82–2.41)	1.50 (0.87–2.56)	1.77 (1.03–3.05)	1.92 (1.11–3.32)	0.02	0.72
≥ 2,400 kcal	1 (Reference)	1.09 (0.84–1.42)	1.12 (0.86–1.47)	1.08 (0.82–1.42)	1.26 (0.96–1.68)	0.16		1 (Reference)	1.53 (0.95–2.46)	0.87 (0.51–1.54)	1.53 (0.93–2.52)	1.14 (0.65–1.99)	0.71	
Waist circumference <85cm	1 (Reference)	1.09 (0.86–1.40)	1.08 (0.84–1.39)	1.10 (0.85–1.43)	1.26 (0.98–1.62)	0.11	0.65	1 (Reference)	1.14 (0.71–1.84)	1.05 (0.64–1.72)	1.53 (0.96–2.46)	1.38 (0.85–2.25)	0.09	0.96
≥ 85cm	1 (Reference)	1.14 (0.84–1.54)	1.21 (0.90–1.63)	1.02 (0.75–1.39)	1.12 (0.81–1.55)	0.79		1 (Reference)	1.21 (0.71–2.08)	1.02 (0.58–1.78)	1.35 (0.79–2.28)	1.33 (0.75–2.36)	0.30	
BMI <24 kg/m^2^	1 (Reference)	1.21 (0.94–1.55)	1.12 (0.87–1.44)	1.11 (0.87–1.41)	1.07 (0.83–1.37)	0.15	0.99	1 (Reference)	1.30 (0.80–2.11)	1.17 (0.71–1.91)	1.60 (0.99–2.59)	1.39 (0.84–2.31)	0.14	0.73
≥24 kg/m^2^	1 (Reference)	1.00 (0.75–1.34)	0.93 (0.68–1.26)	0.98 (0.71–1.35)	0.99 (0.71–1.40)	0.90		1 (Reference)	1.44 (0.86–2.43)	1.16 (0.66–2.04)	1.38 (0.78–2.44)	1.52 (0.85–2.74)	0.27	
Physical activity < 200 (METs–hour/week)	1 (Reference)	1.20 (0.91–1.57)	1.00 (0.74–1.34)	1.06 (0.76–1.48)	1.12 (0.78–1.62)	0.83	0.42	1 (Reference)	1.67 (0.99–2.81)	1.49 (0.86–2.56)	1.75 (0.98–3.13)	1.49 (0.75–2.94)	0.19	0.59
≥ 200 (METs–hour/week)	1 (Reference)	0.91 (0.70–1.18)	1.06 (0.82–1.36)	1.03 (0.80–1.33)	1.10 (0.86–1.41)	0.23		1 (Reference)	1.14 (0.70–1.84)	0.99 (0.60–1.63)	1.37 (0.85–2.20)	1.40 (0.87–2.24)	0.09	
Smoking status Never	1 (Reference)	1.16 (0.85–1.56)	1.13 (0.82–1.54)	1.05 (0.76–1.46)	1.20 (0.86–1.68)	0.48	0.51	1 (Reference)	1.96 (1.02–3.78)	1.72 (0.85–3.46)	1.72 (0.85–3.49)	2.05 (1.00–4.22)	0.17	0.36
Ever	1 (Reference)	1.00 (0.78–1.27)	1.00 (0.79–1.27)	1.08 (0.84–1.38)	1.10 (0.85–1.42)	0.36		1 (Reference)	1.23 (0.80–1.89)	1.05 (0.68–1.63)	1.49 (0.97–2.30)	1.34 (0.85–2.12)	0.13	
Alcohol drinking status Abstainer	1 (Reference)	1.11 (0.80–1.54)	1.00 (0.71–1.41)	1.14 (0.81–1.59)	1.02 (0.72–1.45)	0.90	0.54	1 (Reference)	1.88 (0.97–3.63)	1.21 (0.58–2.54)	1.91 (0.96–3.81)	1.80 (0.88–3.68)	0.19	0.75
Drinker	1 (Reference)	1.01 (0.80–1.28)	1.07 (0.85–1.35)	1.02 (0.79–1.30)	1.21 (0.95–1.55)	0.19		1 (Reference)	1.22 (0.79–1.86)	1.20 (0.79–1.84)	1.42 (0.92–2.21)	1.42 (0.90–2.23)	0.14	

*Hazard ratios and 95% CIs are adjusted for age, urban or rural location, education, waist circumference, ever smoking (never, ever), alcohol drinking (abstainer or drinker), personal income, physical activity, and energy intake*.

*P_trend_, p for trend; P_interaction_, p for interaction*.

## Discussion

Over the past two decades, with the rapid economic growth, the food consumption patterns and dietary behavior of Chinese residents have undergone great changes (30). Nutrition-related noncommunicable diseases, including obesity, type 2 diabetes, hypertension, cardiovascular disease, and some tumor epidemics, continue to challenge the Asian health sector (31–33). This study used data from CHNS 2000–2011 to explore the relationship between carbohydrate intake and new-onset hypertension and subtypes in Chinese adults. In this study, the age-adjusted incidence rate of HTN was 4.86 (95% CI, 4.68–5.04)/100 person-years, and the age-adjusted incidence rates of ISH, IDH, and SDH were 1.48 (95% CI, 1.38–1.59), 1.69 (95% CI, 1.59–1.80) and 1.38 (95% CI, 1.28-1.47)/100 person-years, respectively. The incidence of SDH increased with increasing carbohydrate intake in both men and women. This study revealed that high carbohydrate intake was related to a positive impact on SDH, and we found no relationship between carbohydrate intake and HTN, ISH, and IDH.

This study additionally found that during the mean follow-up period of 6.9 person-years, the age-adjusted HTN incidence was 4.86 (95% CI 4.68-5.04)/100 person-years, similar to previous CHNS study data (9, 34), higher than Canadian residents (3.21/100 years) (35), and higher than the incidence rate of residents in the Middle East (3.36/100 person-years) (36). Furthermore, we found that the incidence of ISH was also higher than in Taiwan (37) (1.48 vs. 1.18/100 person-years), and the incidence of IDH was more than twice that in Taiwan (1.69 vs. 0.63/100 person-years). Our study found that the age-adjusted incidence rate of SDH was 1.38/100 person-years, higher than a previous study (rough incidence rate of 1.1/100 person-years) (9) and the Middle East (36) (crude incidence rate of 0.63/100 person-years). The difference of the results may be related to the difference of the study population, follow-up time and the different lifestyles, mainly in the dietary habits.

This study shows that age-adjusted incidence of SDH increased with the increase of carbohydrate intake. At present, there is still much controversy about the relationship between carbohydrate intake and the incidence of hypertension. Carbohydrates are the main source of energy in the diet in China. High intake of carbohydrates and low intake of fat and protein were associated with insulin resistance and hypertension, possibly by increasing inflammatory factors in the rural population of China (15). Several studies have demonstrated positive associations between dietary carbohydrate intake and HTN (15, 38, 39), but these studies were mainly concerned with the relationship between daily intake of carbohydrates and hypertension, blood pressure, and most of them were cross-sectional studies. Appel et al. found that in prehypertension or hypertensive patients, partial replacement of carbohydrates with protein or monounsaturated fat can further lower blood pressure (17, 40), thus reducing carbohydrate intake rather than increasing protein, or monounsaturated fat intake may be a dietary factor that lowers blood pressure. Systematic reviews and meta-analyses have shown that high carbohydrate intake is associated with a significant increase in blood pressure (41, 42). However, there are still some inconsistent conclusions. Lelong et al. found that carbohydrates were not associated with the risk of hypertension, but the mean follow-up time of that study was only 3.4 years (16). Another cohort study of middle-aged men found that total carbohydrate intake was inversely and significantly related to an average annual change in systolic pressure (43). However, the dietary assessment method for that study varied from our study.

The relationship between carbohydrate intake and hypertension was different in men and women subgroups, considering that sex differences in SDH can be explained by the influence of sex hormone types, dietary intake and environment. Recent studies have shown that there were gender differences in the relationship between dietary factors and the risk of chronic diseases such as hypertension (44, 45). Some studies have shown that the link between carbohydrate intake and metabolic diseases is stronger in women (46). Further studies will be required in the future to investigate the relationship between these factors and the incidence of ISH and IDH.

Studying the relationship between carbohydrate intake and the incidence of hypertension is of great significance for the prevention and treatment of hypertension in China. A deeper understanding of these relations may elucidate novel lifestyle approaches to prevent HTN. Understanding the specific effects of carbohydrate nutrition on BP will help people to facilitate a personalized and targeted lifestyle that addresses the support optimal BP control. Since more individuals now meet the criteria for prehypertension and HTN according to the new BP guidelines, such diet management is likely to have a high public health impact. Therefore, given the changes in dietary nutrition and related epidemics, the government needs to take immediate action to implement effective interventions to promote healthy diets and reduce the burden of chronic non-communicable diseases.

The advantages of this study include a prospective design, a large sample size, and control of a high number of potential confounders. Additionally, this is the first study in China on the relationship between carbohydrate intake and the subtypes of hypertension. Our study has potential limitations. First, only baseline levels of carbohydrate intake are considered and dietary changes may occur during follow-up. However, even if significant dietary changes occur after baseline assessment, they may weaken the observed connections. Furthermore, the number of follow-up participants gradually increased in the cohort study, which will have a certain impact on the representation of people of all ages. In addition, levels of dietary intake (e.g. solid or liquid carbohydrates, whole or refined grains, sodium, and crude fiber intake) were estimated based on 24-hour dietary recalls, which might not have representativeness of a subject's typical intake. Therefore, this cohort study did not analyze the association between carbohydrates types and hypertension.

## Conclusions

The incidence of SDH increased with the increase of carbohydrate energy intake, which was not observed in HTN, ISH, and IDH in men and women. High carbohydrate consumption was related to a positive impact on SDH, but there were no similar associations between carbohydrate intake and new-onset HTN, ISH, and IDH in male and female Chinese adults.

## Data Availability Statement

The raw data supporting the conclusions of this article will be made available by the authors, without undue reservation.

## Author Contributions

All authors listed have made a substantial, direct, and intellectual contribution to the work and approved it for publication.

## Funding

This study was funded by the National Natural Science Foundation of China (81172666), Institute of Nutrition and Health, Chinese Center for Disease Control and Prevention, China, Caroline Population Center, the University of North Carolina at Chapel Hill, National Institutes of Health (NIH) (R01-HD30880, DK056350, R24HD050924, and R01-HD38700), and Fogarty International Center, NIH. This study was funded by the medical science research project plan of Hebei province in 2019 (20190037) and School project of Hebei medical university (CYQD201823).

## Conflict of Interest

The authors declare that the research was conducted in the absence of any commercial or financial relationships that could be construed as a potential conflict of interest.

## Publisher's Note

All claims expressed in this article are solely those of the authors and do not necessarily represent those of their affiliated organizations, or those of the publisher, the editors and the reviewers. Any product that may be evaluated in this article, or claim that may be made by its manufacturer, is not guaranteed or endorsed by the publisher.
